# Supplementary material for: The potential of a universal influenza virus-like particle vaccine expressing a chimeric cytokine
Source: Life Sci Alliance. 2022 Nov 7;6(1):e202201548. doi: 10.26508/lsa.202201548 (PMC9644419; doi:10.26508/lsa.202201548)
Supplement: Supplemental Data 1. [file LSA-2022-01548_Supplemental_Data_1.pdf]

[GENETYX-MAC: Translation of Nucleotides into Amino Acids for Thesis]
Date   : 2018.08.16
Filename   : MP2-NAstalk-murineIL12p40-35-addLinkerGG_0814.seq
Sequence size   : 2136
Sequence Position: 1 - 2136

Translation Position: 1 -   2136

Genetic Code: Standard Genetic Code

```
        10        20        30        40        50        60
ATGTCACTGCTGACAGAAGTGGAAACACCTATCAGAAACGAATGGGGTTGCAGATGCAAC
M  S  L  L  T  E  V  E  T  P  I  R  N  E  W  G  C  R  C  N
A/Aichi/2/1968 (H3N2) MP2 conserved region (Human consensus)

        70        80        90       100       110       120
GACTCATCAGACCCTCTGGTGGTGGCTGCTTCAATCATCGGTATCCTGCACCTGATCCTG
D  S  S  D  P  L  V  V  A  A  S  I  I  G  I  L  H  L  I  L
            Transmembrane domain

       130       140       150       160       170       180
TGGATCCTGGACAGACTGTTCTTCAAATGCATCTACAGATTCTTCGAACACGGTCTGAAA
W  I  L  D  R  L  F  F  K  C  I  Y  R  F  F  E  H  G  L  K


       190       200       210       220       230       240
AGAGGTCCTTCAACAGAAGGTGTGCCTGAATCAATGAGAGAAGAATACAGAAAAGAACAA
R  G  P  S  T  E  G  V  P  E  S  M  R  E  E  Y  R  K  E  Q


       250       260       270       280       290       300
CAATCAGCTGTGGACGCTGACGACTCACACTTCGTGTCAATCGAACTGGAAGGTGACTAC
Q  S  A  V  D  A  D  D  S  H  F  V  S  I  E  L  E  G  D  Y
                                                Linker G

       310       320       330       340       350       360
AAAGACGACGACGACAAAGGTGGTATGAACCCTAACCAAAAAATCATCACAATCGGTTCA
K  D  D  D  D  K  G  G  M  N  P  N  Q  K  I  I  T  I  G  S
Flag sequence  Linker GG N2 cytoplasmic tail Transmembrane domain

       370       380       390       400       410       420
GTGTCACTGACAATCGCTACAGTGTGCTTCCTGATGCAAATCGCTATCCTGGTGACAACA
V  S  L  T  I  A  T  V  C  F  L  M  Q  I  A  I  L  V  T  T
A/Aichi/2/1968(H3N2) N2NA

       430       440       450       460       470       480
GTGACACTGCACTTCAAACAATACGAATGCGACTCACCTGCTTCAAACCAAGTGATGCCT
V  T  L  H  F  K  Q  Y  E  C  D  S  P  A  S  N  Q  V  M  P
               A/Aichi/2/1968(H3N2) N2NA Stalk region

       490       500       510       520       530       540
TGCGAACCTATCATCATCGAAAGAAACATCACAGAAATCGTGTACCTGAACAACACAACA
C  E  P  I  I  I  E  R  N  I  T  E  I  V  Y  L  N  N  T  T
           A/Aichi/2/1968(H3N2) N2NA Stalk region

       550       560       570       580       590       600
ATCGAAAAAGAAGGTGGTATGTGGGAACTGGAAAAAGACGTGTACGTGGTGGAAGTGGAC
I  E  K  E  G  G  M  W  E  L  E  K  D  V  Y  V  V  E  V  D
        Linker GG murine IL12 p40

       610       620       630       640       650       660
TGGACACCTGACGCTCCTGGTGAAACAGTGAACCTGACATGCGACACACCTGAAGAAGAC
W  T  P  D  A  P  G  E  T  V  N  L  T  C  D  T  P  E  E  D


       670       680       690       700       710       720
GACATCACATGGACATCAGACCAAAGACACGGTGTGATCGGTTCAGGTAAAACACTGACA
D  I  T  W  T  S  D  Q  R  H  G  V  I  G  S  G  K  T  L  T
```

```
       730       740       750       760       770       780
ATCACAGTGAAAGAATTCCTGGACGCTGGTCAATACACATGCCACAAAGGTGGTGAAACA
I  T  V  K  E  F  L  D  A  G  Q  Y  T  C  H  K  G  G  E  T

       790       800       810       820       830       840
CTGTCACACTCACACCTGCTGCTGCACAAAAAAGAAAACGGTATCTGGTCAACAGAAATC
L  S  H  S  H  L  L  L  H  K  K  E  N  G  I  W  S  T  E  I

       850       860       870       880       890       900
CTGAAAAACTTCAAAAACAAAACATTCCTGAAATGCGAAGCTCCTAACTACTCAGGTAGA
L  K  N  F  K  N  K  T  F  L  K  C  E  A  P  N  Y  S  G  R

       910       920       930       940       950       960
TTCACATGCTCATGGCTGGTGCAAAGAAACATGGACCTGAAATTCAACATCAAATCATCA
F  T  C  S  W  L  V  Q  R  N  M  D  L  K  F  N  I  K  S  S

       970       980       990      1000      1010      1020
TCATCATCACCTGACTCAAGAGCTGTGACATGCGGTACAGCTTCACTGTCAGCTGAAAAA
S  S  S  P  D  S  R  A  V  T  C  G  T  A  S  L  S  A  E  K

      1030      1040      1050      1060      1070      1080
GTGACACTGGACCAAAGAGACTACGAAAAATACTCAGTGTCATGCCAAGAAGACGTGACA
V  T  L  D  Q  R  D  Y  E  K  Y  S  V  S  C  Q  E  D  V  T

      1090      1100      1110      1120      1130      1140
TGCCCTACAGCTGAAGAAACACTGCCTATCGAACTGGCTCTGGAAGCTAGACAACAAAAC
C  P  T  A  E  E  T  L  P  I  E  L  A  L  E  A  R  Q  Q  N

      1150      1160      1170      1180      1190      1200
AAATACGAAAACTACTCAACATCATTCTTCATCAGAGACATCATCAAACCTGACCCTCCT
K  Y  E  N  Y  S  T  S  F  F  I  R  D  I  I  K  P  D  P  P

      1210      1220      1230      1240      1250      1260
AAAAACCTGCAAATGAAACCTCTGAAAAACTCACAAGTGGAAGTGTCATGGGAATACCCT
K  N  L  Q  M  K  P  L  K  N  S  Q  V  E  V  S  W  E  Y  P

      1270      1280      1290      1300      1310      1320
GACTCATGGTCAACACCTCACTCATACTTCTCACTGAAATTCTTCGTGAGAATCCAAAGA
D  S  W  S  T  P  H  S  Y  F  S  L  K  F  F  V  R  I  Q  R

      1330      1340      1350      1360      1370      1380
AAAAAAGAAAAAATGAAAGAAACAGAAGAAGGTTGCAACCAAAAAGGTGCTCTGCTGGTG
K  K  E  K  M  K  E  T  E  E  G  C  N  Q  K  G  A  L  L  V

      1390      1400      1410      1420      1430      1440
GAAAAAACATCAACAGAAGTGCAATGCAAAGGTGGTAACGTGTGCGTGCAAGCTCAAGAC
E  K  T  S  T  E  V  Q  C  K  G  G  N  V  C  V  Q  A  Q  D

      1450      1460      1470      1480      1490      1500
AGATACTACAACTCATCATGCTCAAAATGGGCTTGCGTGCCTTGCAGAGTGAGATCAGGT
R  Y  Y  N  S  S  C  S  K  W  A  C  V  P  C  R  V  R  S  G

      1510      1520      1530      1540      1550      1560
GGTGGTGGTACAGGTGGTGGTGGTTCAGGTGGTGGTGGTACAGGTGGTGGTGGTAGAGTG
G  G  G  T  G  G  G  G  S  G  G  G  G  T  G  G  G  G  R  V
Linker sequence
```

```
      1570      1580      1590      1600      1610      1620
ATCCCTGTGTCAGGTCCTGCTAGATGCCTGTCACAATCAAGAAACCTGCTGAAAACAACA
I  P  V  S  G  P  A  R  C  L  S  Q  S  R  N  L  L  K  T  T
murine IL12 p35

      1630      1640      1650      1660      1670      1680
GACGACATGGTGAAAACAGCTAGAGAAAAACTGAAACACTACTCATGCACAGCTGAAGAC
D  D  M  V  K  T  A  R  E  K  L  K  H  Y  S  C  T  A  E  D

      1690      1700      1710      1720      1730      1740
ATCGACCACGAAGACATCACAAGAGACCAAACATCAACACTGAAAACATGCCTGCCTCTG
I  D  H  E  D  I  T  R  D  Q  T  S  T  L  K  T  C  L  P  L

      1750      1760      1770      1780      1790      1800
GAACTGCACAAAAACGAATCATGCCTGGCTACAAGAGAAACATCATCAACAACAAGAGGT
E  L  H  K  N  E  S  C  L  A  T  R  E  T  S  S  T  T  R  G

      1810      1820      1830      1840      1850      1860
TCATGCCTGCCTCCTCAAAAAACATCACTGATGATGACACTGTGCCTGGGTTCAATCTAC
S  C  L  P  P  Q  K  T  S  L  M  M  T  L  C  L  G  S  I  Y

      1870      1880      1890      1900      1910      1920
GAAGACCTGAAAATGTACCAAACAGAATTCCAAGCTATCAACGCTGCTCTGCAAAACCAC
E  D  L  K  M  Y  Q  T  E  F  Q  A  I  N  A  A  L  Q  N  H

      1930      1940      1950      1960      1970      1980
AACCACCAACAAATCATCCTGGACAAAGGTATGCTGGTGGCTATCGACGAACTGATGCAA
N  H  Q  Q  I  I  L  D  K  G  M  L  V  A  I  D  E  L  M  Q

      1990      2000      2010      2020      2030      2040
TCACTGAACCACAACGGTGAAACACTGAGACAAAAACCTCCTGTGGGTGAAGCTGACCCT
S  L  N  H  N  G  E  T  L  R  Q  K  P  P  V  G  E  A  D  P

      2050      2060      2070      2080      2090      2100
TACAGAGTGAAAATGAAACTGTGCATCCTGCTGCACGCTTTCTCAACAAGAGTGGTGACA
Y  R  V  K  M  K  L  C  I  L  L  H  A  F  S  T  R  V  V  T

      2110      2120      2130
ATCAACAGAGTGATGGGTTACCTGTCATCAGCTtaa
I  N  R  V  M  G  Y  L  S  S  A  *
```
