# Supplementary material for: The potential of a universal influenza virus-like particle vaccine expressing a chimeric cytokine
Source: Life Sci Alliance. 2022 Nov 7;6(1):e202201548. doi: 10.26508/lsa.202201548 (PMC9644419; doi:10.26508/lsa.202201548)
Supplement: Supplementary file 2 [file LSA-2022-01548_SdataF2.pdf]

Uncropped images of western blotting in Fig. 2

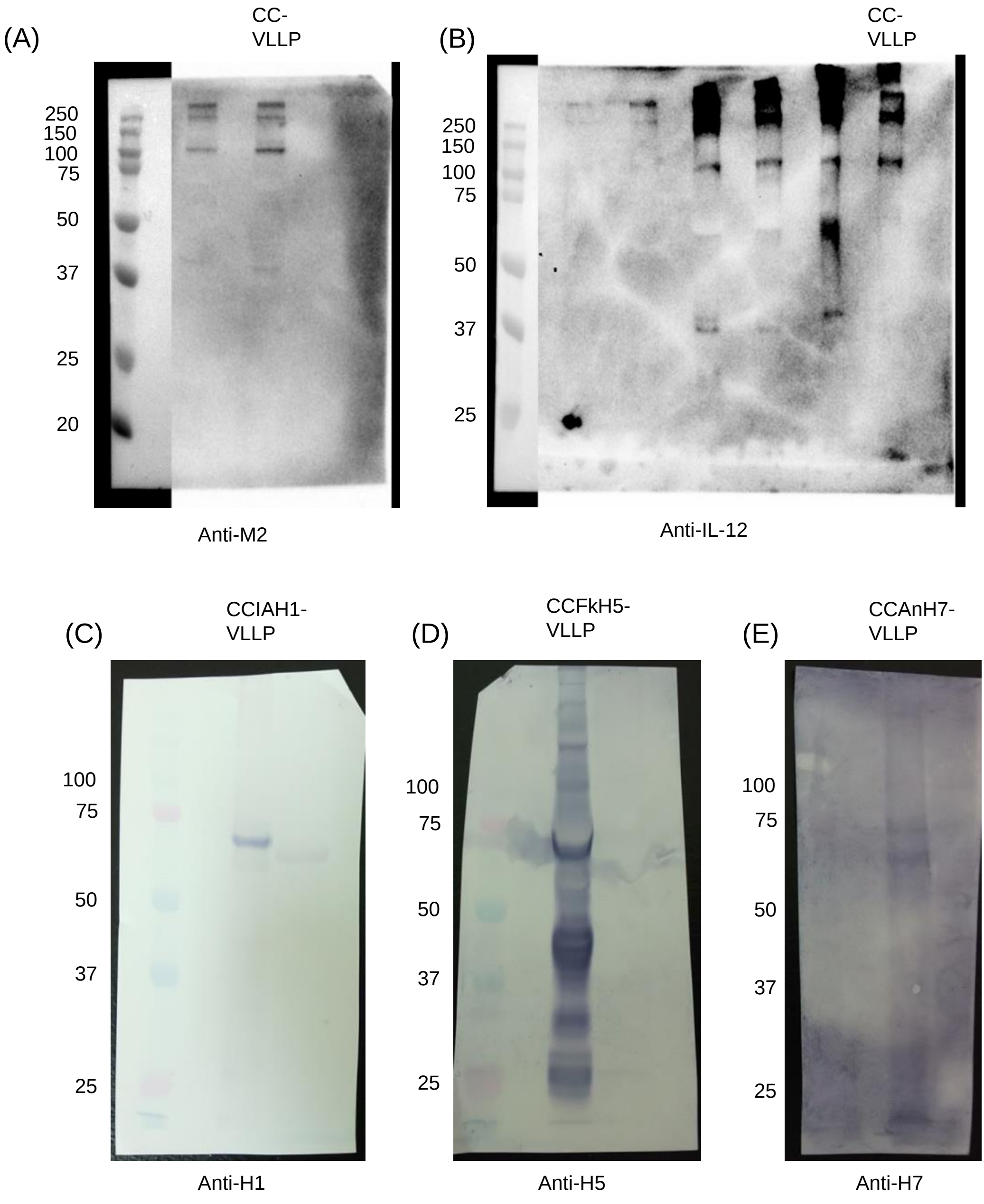

Uncropped images of western blotting are shown. Images A-E correspond to those in Fig. 2. The sample name indicates the representative lane in M2 and IL-12 images.
