# Supplementary material for: The potential of a universal influenza virus-like particle vaccine expressing a chimeric cytokine
Source: Life Sci Alliance. 2022 Nov 7;6(1):e202201548. doi: 10.26508/lsa.202201548 (PMC9644419; doi:10.26508/lsa.202201548)
Supplement: Supplementary file 4 [file LSA-2022-01548_TableS1.docx]

Table S1. Accession No. of strains used for constructing the phylogenetic tree in Fig. 10

| **HA subtype** | **Strain** | **Accession number** |
| --- | --- | --- |
| H1 | A/Taiwan/4845/1999(H1N1) | DQ415318 |
| H2 | A/Berlin/3/1964(H2N2) | L11126 |
| H3 | A/Taiwan/220/2004(H3N2) | DQ415325 |
| H4 | A/mule duck/Bulgaria/105/2008(mixed) clone H4 | KP714468 |
| H5 | A/duck/Vietnam/NCVD-0004/2013(H5N1) | KY171536 |
| H6 | A/Mallard duck/Alberta/520/2019(H6N5) | MT624504 |
| H7 | A/ruddy turnstone/Delaware Bay/66/2020(H7N3) | MW876037 |
| H8 | A/mallard/Alberta/48/2017(H8N4) | MH412114 |
| H9 | A/Ruddy Turnstone/Delaware/418/2019(H9N2) | MN908074 |
| H10 | A/Sanderling/Delaware/510/2021(H10N9) | OM966092 |
| H11 | A/Ruddy Turnstone/Delaware Bay/447/2020(H11N9) | MW875147 |
| H12 | A/ruddy turnstone/Delaware Bay/301/2020(H12N9) | MW875537 |
| H13 | A/Red Knot/Delaware Bay/605/2020(H13N6) | MW874987 |
| H14 | A/long-tailed duck/Wisconsin/10OS4225/2010(H14N6) | JN696316 |
| H15 | A/mallard/Novomychalivka/2-23-12/2010(H15N7) | KP087869 |
| H16 | A/Laughing Gull/Delaware/583/2021(H16N3) | OM965805 |
| B/Yamagawa | B/Minnesota/15/2020 | MT423089 |
| B/Victoria | B/Colorado/01/2019 | MK715543 |
| B/Lee | B/Lee/40 | DQ792897 |
